# Effect of the Chemical and Mechanical Recycling of PET on the Thermal and Mechanical Response of Mortars and Premixed Screeds

**DOI:** 10.3390/ma16083155

**Published:** 2023-04-17

**Authors:** Michela Lerna, Dora Foti, Andrea Petrella, Maria Francesca Sabbà, Sulyman Mansour

**Affiliations:** 1 Department of Architecture, Construction and Design (DArCoD), Polytechnic University of Bari, 70125 Bari, Italy; 2 Department of Civil, Environmental, Land, Construction and Chemistry, Polytechnic University of Bari, 70125 Bari, Italy

**Keywords:** PET-recycling, plastic waste, depolymerized PET (DPET), thermal, mechanical properties

## Abstract

In this paper, recycled polyethylene terephthalate (PET) was used to produce eco-innovative engineering materials with optimized performance, minimizing the environmental impact deriving from plastic consumption activity and limiting the continuous consumption of raw materials. The recycled PET obtained from waste bottles, commonly used to improve the ductility of concrete, has been used with a different weight percentage as plastic aggregate in the replacement of sand in cement mortars and as fibers added to premixed screeds. In detail, the effect of PET treatment (chemical or mechanical) on the thermal performance was evaluated. Non-destructive physical tests were conducted to determine the thermal conductivity of the investigated building materials. The performed tests showed that chemically depolymerized PET aggregate and recycled PET fibers derived from plastic wastes can reduce the heat conduction capacity of the cementitious materials with limited reduction in compressive strength. The results of the experimental campaign have made it possible to evaluate the influence of the recycled material on the physical and mechanical properties and its feasibility in non-structural applications.

## 1. Introduction

Plastics are classified among solid waste generated in greater quantities and represent a significant threat to environmental sustainability. Plastic has become an integral and essential product in the consumption process due to certain characteristics which make it a highly competitive material in the marketplace: strength and low density, high moldability, durability, biological and chemical inertness, electrical and thermal insulator, and low cost [1]. In fact, plastic materials are used in various fields of application, i.e., packaging, car and industrial applications, construction sector, medical equipment, other health applications, and food distribution. For this reason, plastic materials contribute to the ever-growing volume of the flow of produced solid waste. More than 350 million tons of plastics are being produced worldwide annually in various applications, including packaging, building and construction, textiles, consumer and institutional products, transportation, electrical and electronic equipment, and industrial machinery. Although plastics are valuable resources in many aspects, the proliferation of plastic products in the last several decades has resulted in a negative environmental footprint due to poor recycling rates after first use. Despite this obvious problem, plastic production volume is expected to continuously increase over the next few decades. Currently, about 70% of global plastics are found as waste. Only around 41% of post-consumer plastic waste is recovered by recycling and incineration with an energy generation process, whereas 40% is disposed in landfills and 19% ends up in the oceans or on coastlines [2]. In addition, the low biodegradability of plastic places a limit on the quantity of refusal that could potentially be disposed, as well as on the recyclability process. Based on these aspects, the research focuses on reducing consumption sources and enhancing its reuse and recycling in order to limit the amount of plastic generated every year in the world and slow down the continuous increase in the production of plastic. Therefore, finding effective alternative recycling methods for waste can help to guarantee a sustainable environment by preventing contamination and allowing us to obtain eco-suitable solutions in the field of civil constructions. In 2015, the United Nations launched the sustainable development objectives (SDG) which should be capable of guaranteeing environmental protection by 2030, developing sustainable cities in the process [3,4,5]. In this context, the replacement of traditional construction materials with plastic products obtained by recycled waste is a sustainable solution that mitigates the overcrowding of landfills and reduces the use of raw materials [6].

Polyethylene terephthalate (PET) waste obtained from packaging and mineral water bottles have been used as alternative/additional materials in the construction sector. The practical available methods for PET recycling are primary recycling, mechanical or secondary recycling, chemical or tertiary recycling, and energy recovery through incineration recycling [7,8,9,10,11]. Mechanical and chemical processes have been used to produce recycled PET that, when introduced in construction materials, improves their physical and mechanical properties. Mechanical recycling is the ideal method when applied to a source separated from plastic waste and it is preferred as the most suitable method of recycling plastics over melting, reshaping, and other chemical methods since the reduction in mechanical properties is minimal [6]. In general, chemical recycling with a depolymerization reaction leads to mechanical and thermal property changes [8]. PET used in bottles can be used to produce fibers by a mechanical process which is a simple, cost-effective, and environmentally friendly process. Recycled PET fibers can be cut and used to reinforce concrete with a high potentiality of placing other virgin fibers. In fact, the literature has shown that PET fibers contained in the mixture improve the mechanical (flexural strength, ductility, and unconfined compressive strength) and physical properties of concrete and prevent (or delay considerably) the cracking process. To produce a low-cost composite material that contributes to the sustainable concrete sector, prior researchers examined the inclusion of PET fibers in concretes and mortars [8,9,10,11,12,13,14,15]. De Oliveira et al., in [16], presented an experimental campaign conducted on eco-efficient mortars in order to evaluate the physical and mechanical properties and toughness indices of PET fiber mortars reinforced with different volumes of fibers (0%, 0.5%, 1.0%, and 1.5%). The results reported in [16] show that the density of hardened the mortar is not significantly altered by the incorporation of PET fibers, just as it does not significantly change the mortar’s compressive strength. Similar results on compressive strength are found in [12,17]. More widely, studies tend to be concerned with the mechanical behavior of recycled PET concrete, particularly the flexural strength increase that is obtainable through the use of recycled reinforcement [14,18,19,20].

A further use of recycled PET waste is its use as an additive in asphalt, cementitious materials, mortars, or concrete. Depolymerized PET (DPET) aggregates, obtained by using chemical recycling methods in monomers, are used in the production of construction materials to improve the mechanical properties of these composites [21,22,23,24,25,26,27,28]. The most frequently applied methods use water (hydrolysis), glycols (glycolysis), amines (aminolysis), and alcohols (alcoholysis) for de-polymerization of PET waste under various reaction conditions [23].

Many researchers have exerted considerable effort to investigate the effect of recycled PET on the properties of mortar and concrete. Their studies have produced different results depending on the type, size, and proportion of plastic. Generally, the density obtained is decreased, but the flexural strength is increased with the use of PET. The slump of the material containing recycled plastic is reduced due to many reasons, such as angular particle size and the sharp edges of plastic aggregate. Low fluidity, which is a result of non-uniform shapes of plastic aggregates, and the large ratio of fine particles or powder (which is often caused by grinding plastic) increases the total surface area, thus increasing the need to increase water content to surround these particles [28]. These characteristics make the use of composite materials in applications feasible, such as in road flooring, underbases for sidewalks, and various structures in which resistance is not a crucial factor and in applications in which a lighter material is useful.

In cementitious materials, thermal conductivity is a crucial factor when considering the amount of heat transfer through conduction. Large amounts of heat are usually lost through roofs and walls and thus affect the energy consumption of buildings. The energy consumption and heat transfer of a building decrease when concrete has a low thermal conductivity. The 15% of the heating energy in European countries can be reduced by using structurally lightweight aggregate concrete instead of using normal weight concrete [28]. Concrete thermal conductivity clearly varies with the substitution level of plastic waste as the aggregate [29,30]. The thermal conductivity of sand is higher than PWA. The global conductivity of the composite decreases as the rate of plastic waste as the aggregate rises. In comparison with sand, PWA slows down thermal heat transfer [28,30,31]. As it emerges in [32], despite the large number of studies that have been conducted on the development of recycled plastic as a reinforcement, several aspects remain uninvestigated in regard to the use of recycled PET in the production of composite materials regarding the properties of thermal insulation. Lazorenko, in [33], analyzed geopolymer mortars, discovering that the use of PET increases thermal insulation properties up to 59% when the replacement level of natural aggregate is equal to 100%. In this work, the results on the thermal properties of two types of materials, mortars and premixed screeds reinforced with DPET and PET fibers, are presented. In more detail, recycled PET, treated through chemical or mechanical method, are introduced in the mixtures as a replacement of fine sand or in addition as fibers. 

## 2. Materials and Test Methods

**Figure 1:**
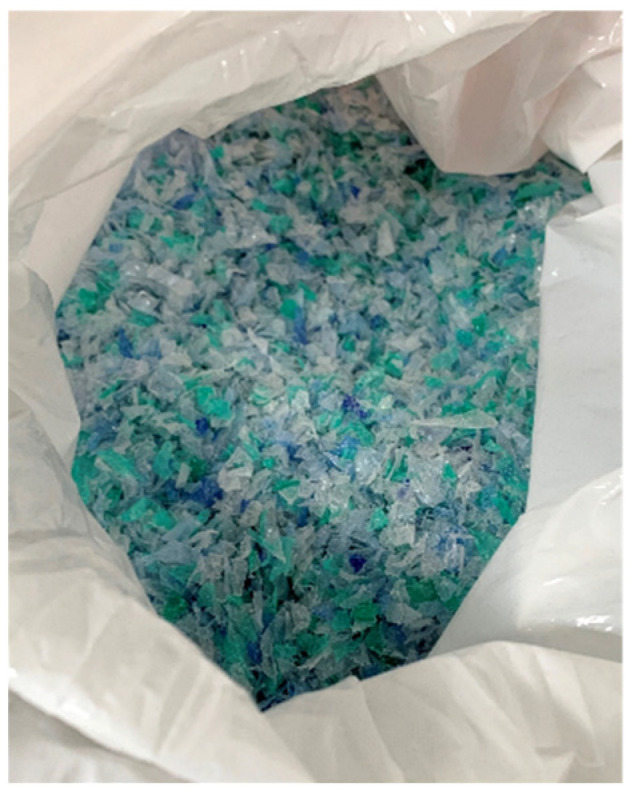
Plastic raw material used for the chemical and mecchanical process.

The experimental program presented in this study aimed to assess the thermal properties and the compressive strength of mortars and screeds for non-structural applications, using different PET-types derived from chemical and mechanical recycling methods of the material in Figure 1. Furthermore, the insulating performance results of the tested material are reported and discussed with respect to commonly adopted reinforcement techniques.

Two mortar mixtures (M2_25 and M3_50) containing DPET additive produced from glycolysis chemical degradation of plastic bottles were tested and compared with a target mortar (M1_0). DPET aggregate was incorporated in the target mortars, replacing different sand weight ratios, i.e., 25% and 50%. Portland cement type CEM I-52.5R and fine sand (0.2–5 mm) was used to produce the mortars and water-cement ratio (W/C) was set equal to 0.45.

Two commercial screeds identified as target groups are investigated in this research. The premixed screed identified with TS acronym is a traditional material used to realize civil backgrounds and flooring, while the premixed screed identified with AS acronym is a material classified as self-levelling. Both target screeds are available as powder and need to be mixed only with water. More information on the composition can be found on the supplier’s website [34]. PET fibers obtained by mechanical cut of plastic bottles were added to the target screeds with a weight ratio equal to 0.50%, 0.75%, and 1.00% by weight of mixture (powder plus water). 

The experimental research programs conducted on the mortars and the premixed screeds are shown in Table 1. In Figure 1, plastic raw material used in the research is shown.

**Figure 2:**
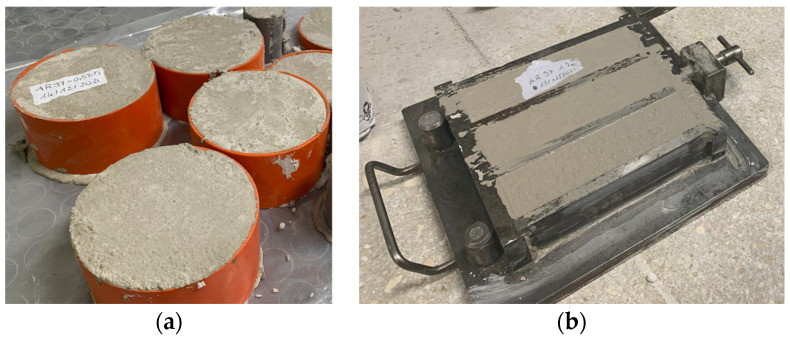
Molding of samples for non-destructive thermal conductivity tests (**a**); molding of samples for compression tests (**b**).

Fresh properties (fresh density and flowability), thermal properties, and compressive strength were investigated for DPET mortars and PET-fiber screeds. Three prismatic specimens were casted into 40 mm × 40 mm × 160 mm steel molds to determine the compression strength; a total of six cubic samples and five specimens were casted into Φ100 mm × h50 mm cylindrical molds to determine the thermal conductivity of the materials (Figure 2). Then, the specimens were treated at a constant temperature and humidity and tested on the 28th day, according to the standard [35]. The details of the design mixtures and replacements/additions of PET adopted in this study are indicated in Table 2. The water/cement ratio adopted to produce the mortars was equal to 0.50. According to the technical specifications of the products [31], the water/powder ratio adopted to produce TS and AS-screed was 0.13 and 0.17, respectively.

The materials were mixed in a planetary mixer according to the recommendations in [32,36]. To produce the mortars, the aggregates were blended for 1 min, then the cement and water were added and mixed with the aggregates for 1 min. Then, PET was added and all materials were mixed for 1 min before casting and starting the tests. 

**Figure 3:**
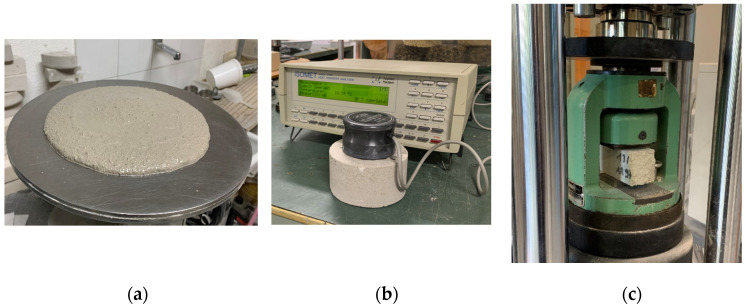
Characterization tests: (**a**) flow table test; (**b**) thermal conductivity test; (**c**) compressive test.

Flow table tests were conducted to determine the flowability of the investigated materials. During the test, the mortars and the screeds were introduced in the truncated conical mold. After approximately 15 s, the mold was slowly raised vertically and the material was spread out on the disc by jolting the flow Table 15 times at a constant frequency of approximately one per second [36]. The flow was measured by checking the diameter of the spread mix, as shown in Figure 3a. In order to evaluate the thermal insulation performance reachable through the investigated reinforcement methods (chemically or mechanically treated PET), the non-destructive thermal conductivity tests were carried out using the device ISOMET 2104 (Applied Precision, Ltd., Bratislava, Slovakia). To this purpose, ϕ = 100 mm, h = 50 mm cylinders were prepared and cured for 28 days. A thermal stress was induced on the sample from a flat source which was placed on the surface. An estimation of the thermal conductivity λ, expressed in W/mK, and of the thermal diffusivity, expressed in m^2^/s, was obtained (Figure 3b). The measurement is based on the analysis of temperature response of the analyzed material to heat flow impulses. The heat flow is induced by electrical heating using a resistor heater having direct thermal contact with the surface of the sample. Furthermore, the analysis of the influence of PET on the compressive strength of the materials was conducted by performing the compressive tests in accordance with [28,37] (Figure 3c). The tests were performed in indoor conditions in M. Salvati Laboratory of Polytechnic University of Bari and the loading rate of the Controls C300 machine was 150 N/sec.

## 3. Results and Discussion

In this section, the results of the tests to determinate the fresh characteristics of the mixes, thermal properties, and compressive strengths are reported and discussed. The main goal of this work is to observe the influence of possible PET applications on the fresh and hard behavior of non-structural materials and to clarify if the performance of the mortars and the screeds, when the treatment of recycled PET is chemical or mechanical type, is more influenced by the effects of the plastic.

### 3.1. Fresh Properties

The fresh density of the investigated materials decreased with a higher content of PET waste, according to the smaller specific gravity of PET, compared to natural sand, as shown in Table 3. A density reduction was found for the investigated mortars with respect to the target values (M1_0) of 7% and 25% for M2_25 and M3_50, respectively. The maximum density reductions found for the premixed screeds were equal to 4% and 2% of the target values TS_0 and AS_0, respectively. The reduction in density was less in the screeds with low fiber content.

**Figure 4:**
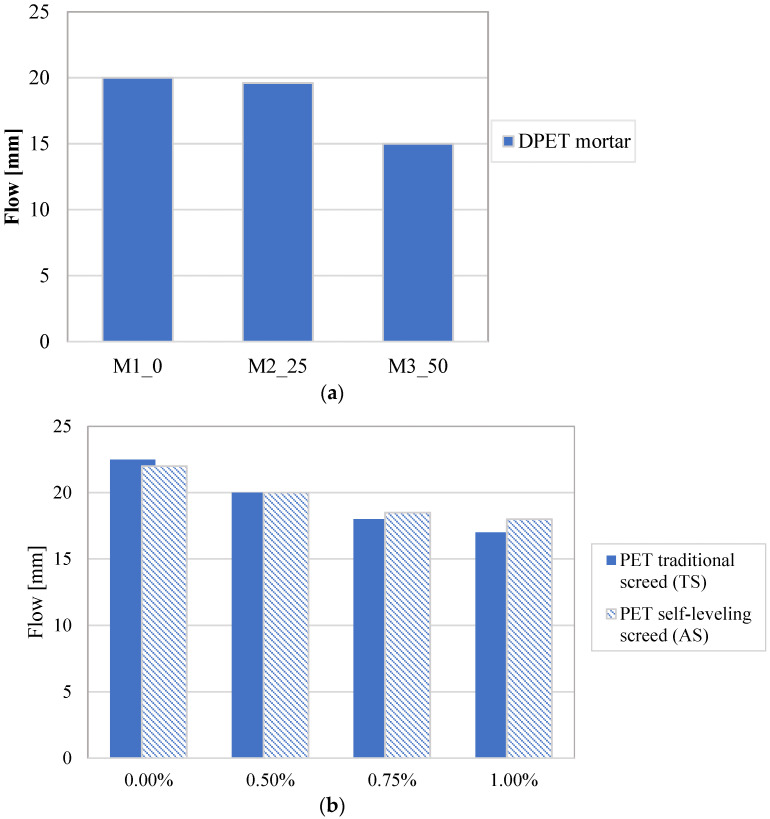
Flow results: (**a**) DPET-mortars; (**b**) PET-fiber screeds.

Figure 4 presents the results of PET-mixture flowability from the flow values obtained through the table tests. Figure 4a shows that the flow value of all DPET mortar samples decreased continuously when the sand replacement increased. The flow variation measured from the PET-fiber screeds is shown in Figure 4b. The maximum flow reduction recorded was equal to 25% for the M3_50 mortar. As for DPET-mortars, the flow value decreased proportionally to the PET-fiber content, obtaining the maximum reduction equal to 22% and 16% for TS_1.00 and TS_1.00, respectively. The self-leveling screeds show a lower workability variation if compared to the tractional screeds reinforced with the same PET-fiber content.

### 3.2. Thermal Properties

The specific weight, thermal conductivity, and thermal diffusivity values obtained for all mixtures are reported in the Table 4. It has been observed that the thermal conductivity value decreased by incorporating ever-increasing PET content (in form of additive or fiber) with respect to the target value. Using the reinforcement technique with chemically treated PET, the thermal conductivity reduction obtained was 35% when the replacement of sand in the mortar was equal to 50%. Adding PET-fibers to the tested screeds, the reduction of thermal conductivity was limited to 35% for the TS traditional screed, while the thermal property of the self-leveling screed was not strongly influenced by the plastic fiber content. The maximum thermal conductivity reduction in AS groups was equal to 9% for AS_0.75 but, in any case, the variation was below 10%.

In Table 4 the mean values of the thermal properties (conductivity and diffusivity) of all tested mixture are represented. From the results it is possible to note that the chemically treated waste PET allowed for better insulation properties. Using the mechanically recycled PET fiber technique, the thermal properties of the material improved, but the thermal conductivity reductions in the AS screed were not relevant for the purpose of a possible insulation application of this material.

### 3.3. Compressive Strength

The compressive strength and the corresponding standard deviation values of the different tested mixtures are collected in Table 5. The compressive strength of DPET mortars decreased by 11%, 36%, 54%, and 59% for DPET additive contents equal to 20%, 40%, 60%, and 80%, respectively. The experimental results show that the compressive strength decreased when DPET content increased. The variation in compressive strength was not proportional to the PET content in accordance with [16,38,39]. From observing the results of the compressive tests conducted on the premixed screeds, it emerges that the strength slightly increased with a percentage the PET-fiber content equal to 0.50% and 0.75%. In any case, the compressive strength of all fiber-reinforced mixtures did not undergo significant variations compared to the target value. The compressive strength of the screeds containing the recycled PET-fiber was comparable to that of the commercial material value.

The incorporation of PET fibers did not significantly change the compressive strength magnitude of the screeds, while the compressive strength of mortars was negatively influenced by DPET content.

**Figure 5:**
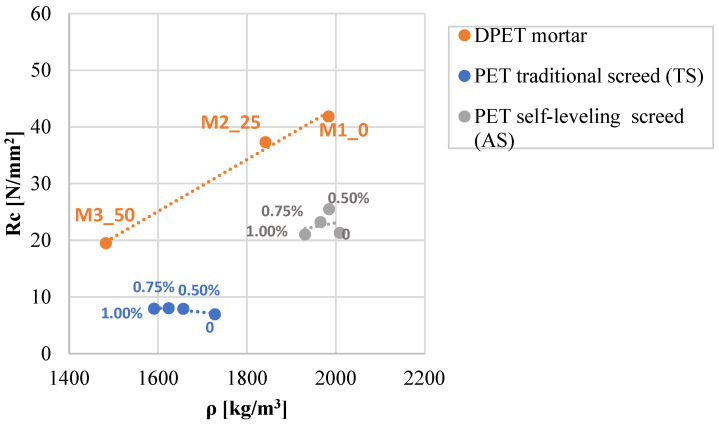
Compressive strength-specific weight relationship of the investigated materials: DPET mortars, PET traditional screeds, and PET self-leveling screeds.

In Figure 5, for all investigated materials, the specific weight decreased when increasing the PET content, but the influence of the waste material on compressive strength depended on the type of PET treatment. The compressive strength of DPET mortars varied linearly with the specific weight, the same relationship was not found in the PET-fiber screeds. The PET fiber traditional screed presented a greater compressive strength, despite having the lowest specific weight.

## 4. Conclusions

The methodologies for the expansion of the PET reuse practice presented and compared in this paper, using mechanical or chemical recycling method of plastic, show the possibility of producing light construction materials. In fact, the use of light additives in the mixture reduces the density of the material while maintaining acceptable levels of workability. This aspect offers many advantages, such as a reduction in the structural dead-weight with a better seismic behavior and the increase in thermal insulation performance of the buildings. 

The compressive strength does not undergo significant reductions for the purpose of applying the material in the non-structural field. In fact, the PET component in the mixture can reduce the compressive strength, but the strength of all samples is maintained at the same grade of the target.

The density variations of the premixed screeds are very limited, consequently the thermal and mechanical measurements, especially in the case of AS-1.0 and AS-0.75 samples, are at the limit of the experimental error. Using the mechanically recycled PET fiber technique, the thermal properties of the material improve, but the reductions in thermal conductivity of the AS screed are not relevant for the purposes of any insulating application of this material. Accordingly, the self-leveling screeds are not strongly influenced by the plastic fiber content. Additionally, for this reason, there are no significant mechanical differences in compression. Indeed, the compressive strength of all fiber-reinforced mixtures does not undergo significant variations with respect to the target value.

In addition, the use of PET waste produces a reduction in pollution with the unquestionable advantages that it would produce.

## Figures and Tables

**Table 1:** Experimental program of PET reinforced materials.

**PET Treatment**	**MORTAR type**		**DPET [%]**
Chemical	Traditional	M1_0	-
		M2_25	25
		M3_50	50
	**SCREED type**		**PET fiber [%]**
Mechanical	Traditional	TS_0	-
		TS_0.50	0.50
		TS_0.75	0.75
		TS_1.0	1.00
	Self-leveling	AS_0	-
		AS_0.50	0.50
		AS_0.75	0.75
		AS_1.00	1.00

**Table 2:** Mix design of the tested mortars and screeds.

**MORTAR**	**Sand [g]**	**Water [mL]**	**DPET [g]**
M1_0	500	225	-
M2_25	375	225	150
M3_50	250	225	237.5
**SCREED**	**Screed powder [g]**	**Water [mL]**	**PET fiber [g]**
TS_0	500	65	/
TS_0.50	500	65	2.29
TS_0.75	500	65	4.24
TS_1.0	500	65	5.65
AS_0	500	65	/
AS_0.50	500	65	2.29
AS_0.75	500	65	4.24
AS_1.00	500	65	5.65

**Table 3:** Fresh density values of the tested mortars and screeds.

**MORTAR**	**DPET [%]**	**Fresh Density [kg/m^3^]**
M1_0	-	2011.7
M2_25	25	1872.3
M3_50	50	1526.8
**SCREED**	**PET fiber [%]**	**Fresh density [kg/m^3^]**
TS_0	/	2046.1
TS_0.50	0.50	2044.1
TS_0.75	0.75	2034.8
TS_1.0	1.00	1982.7
AS_0	/	2198.2
AS_0.50	0.50	2193.3
AS_0.75	0.75	2173.9
AS_1.00	1.00	2174.9

**Table 4:** Specific weight, thermal conductivity, and diffusivity values of DPET mortars and PET-fiber screeds investigated.

**Waste Type**	**MORTAR**	**Specific Weight [kg/m^3^]**	**Thermal Conductivity [W/mK]**	**Thermal Diffusivity [m^2^/s] × 10^−8^**	**Thermal Conductivity Reduction**
**DPET**	M1_0	1984	0.948	0.633	-
	M2_25	1842	0.685	0.524	17%
	M3_50	1483	0.482	0.411	35%
**Waste Type**	**PREMIXED SCREED**	**Specific weight ρ [kg/m^3^]**	**Thermal Conductivity [W/mK]**	**Thermal Diffusivity [m^2^/s] × 10^−8^**	**Thermal Conductivity reduction**
**PET fiber**	TS-0	1728	0.777	0.526	-
	TS-0.50	1657	0.637	0.449	19%
	TS-0.75	1624	0.579	0.411	26%
	TS-1.0	1591	0.508	0.408	35%
	AS-0	2009	0.793	0.527	-
	AS-0.50	1985	0.745	0.543	7%
	AS-0.75	1966	0.722	0.501	9%
	AS-1.0	1931	0.789	0.510	1%

**Table 5:** Compressive strength results with deviation standard values of all tested specimens containing recycled PET.

**Mortar**	**DPET [%]**	**Compressive Strength** ***R_c_* [N/mm^2^]**	**Compressive Strength** **Reduction**
M1_0	-	41.84 ± 0.67	-
M2_25	25	37.31 ± 0.87	−11%
M3_50	50	19.47 ± 0.60	−54%
**Premixed screed**	**PET fiber [%]**	**Compressive strength** ***R_c_* [N/mm^2^]**	**Compressive strength** **variation**
TS-0	-	6.94 ± 0.41	-
TS-0.50	0.50	7.91 ± 0.50	+13%
TS-0.75	0.75	8.02 ± 0.20	+15%
TS-1.0	1.00	7.92 ± 0.45	+14%
AS-0	-	21.36 ± 0.24	-
AS-0.50	0.50	25.47 ± 0.42	+19%
AS-0.75	0.75	23.20 ± 0.97	+8%
AS-1.0	1.00	21.02 ± 0.83	−2%

## Data Availability

The data used to support this research are available from the corresponding author upon request.
